# Apigenin and Structurally Related Flavonoids Allosterically Potentiate the Function of Human α7-Nicotinic Acetylcholine Receptors Expressed in SH-EP1 Cells

**DOI:** 10.3390/cells10051110

**Published:** 2021-05-05

**Authors:** Waheed Shabbir, Keun-Hang Susan Yang, Bassem Sadek, Murat Oz

**Affiliations:** 1 Department of Medicine, Division of Nephrology and Cellular and Molecular Pharmacology, University of California, San Francisco, CA 94158-2140, USA; waheed.shabbir@ucsf.edu; 2 Department of Biological Sciences, Schmid College of Science and Technology, Chapman University, One University Drive, Orange, CA 92866, USA; kyang@chapman.edu; 3 Department of Pharmacology and Therapeutics, College of Medicine and Health Sciences, UAE University, Al Ain 17666, United Arab Emirates; bassem.sadek@uaeu.ac.ae; 4 Department of Pharmacology and Therapeutics, Faculty of Pharmacy, Kuwait University, Safat 13110, Kuwait

**Keywords:** nicotinic receptors, apigenin, flavonoids, positive allosteric modulator, pain, inflammation, neurodegenerative disorders

## Abstract

Phytochemicals, such as monoterpenes, polyphenols, curcuminoids, and flavonoids, are known to have anti-inflammatory, antioxidant, neuroprotective, and procognitive effects. In this study, the effects of several polyhydroxy flavonoids, as derivatives of differently substituted 5,7-dihydroxy-4*H*-chromen-4-one including apigenin, genistein, luteolin, kaempferol, quercetin, gossypetin, and phloretin with different lipophilicities (cLogP), as well as topological polar surface area (TPSA), were tested for induction of Ca^2+^ transients by α7 human nicotinic acetylcholine (α7 nACh) receptors expressed in SH-EP1 cells. Apigenin (10 μM) caused a significant potentiation of ACh (30 μM)-induced Ca^2+^ transients, but did not affect Ca^2+^ transients induced by high K^+^ (60 mM) containing solutions. Co-application of apigenin with ACh was equally effective as apigenin preincubation. However, the effect of apigenin significantly diminished by increasing ACh concentrations. The flavonoids tested also potentiated α_7_ nACh mediated Ca^2+^ transients with descending potency (highest to lowest) by genistein, gossypetin, kaempferol, luteolin, phloretin, quercetin, and apigenin. The specific binding of α7 nACh receptor antagonist [^125^I]-bungarotoxin remained unchanged in the presence of any of the tested polyhydroxy flavonoids, suggesting that these compounds act as positive allosteric modulators of the α7-nACh receptor in SH-EP1 cells. These findings suggest a clinical potential for these phytochemicals in the treatment of various human diseases from pain to inflammation and neural disease.

## 1. Introduction

Nicotinic acetylcholine (nACh) receptors belong to the ligand-gated ion channel family that includes serotonin type-3, glycine, and γ-aminobutyric acid (GABA)-A receptors. The homomeric α7 nACh receptor subtype is expressed in both central and peripheral nervous systems, as well as non-neuronal cells, and plays an important role in synaptic plasticity and various disease pathologies [1]. Thus, α7-nACh receptors are recognized targets for drug development in several preclinical experimental models of pain, inflammation, neurodegenerative diseases, and psychosis [1,2]. Therefore, chemical entities modulating the function these receptors have clinical significance in treating pain and inflammation, and alleviating several neurodegenerative disorders.

**Figure 1:**
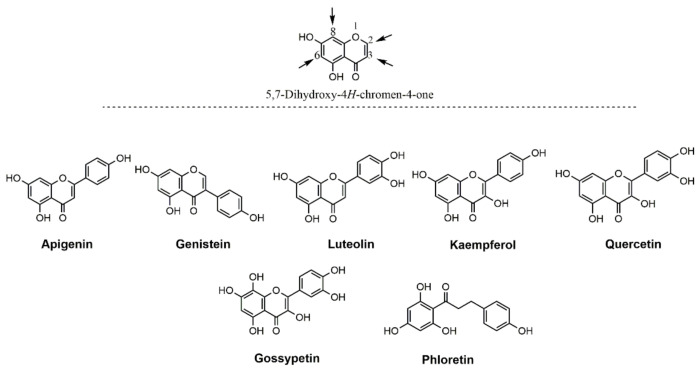
Features similarities between the tested polyphenol flavonoids as derivatives of 5,7-dihydroxy-4H-chromen-4-one.

Phytochemicals, such as terpenes, polyphenols, curcuminoids, and flavonoids, have been shown extensively to exert antioxidant, anti-inflammatory, anti-hypertensive, neuroprotective, antiepileptic, and procognitive effects [3,4,5,6,7]. In search of new compounds, several phytochemicals including terpenes, such as menthol [8], thujone [9], and carveol [10], as well as capsaicin [11], cannabidiol [12], and cannabis terpenes, such as bisabolol [13], have been shown to allosterically modulate the function of α7 nACh receptors in cellular systems. Further studies with more complex phytochemicals identified curcumin and its metabolites as positive allosteric modulators (PAM) of α7 nACh receptor [14,15,16,17]. Importantly, some of the flavonoid-group phytochemicals, such as genistein and quercetin, were recently shown to act as a PAM of α7 nACh receptor [17,18,19]. In the present study, we have investigated the effects of a panel of polyhydroxy flavonoids that are the products of differently substituted 5,7-dihydroxy-4H-chromen-4-one structural skeleton (Figure 1). This includes apigenin, genistein, luteolin, kaempferol, quercetin, gossypetin, and phloretin on human α7-nACh receptors expressed in SH-EP1 cells. In addition, the modulating role of different substituents at the 2-, 3-, 6-, and 8-position of 5,7-dihydroxy-4H-chromen-4-one, the selected compounds on metric parameters were assessed to predictably quantify the lipophilicity (clogP), the water solubility (clogS), and drug-likeness score applying Molinspiration Property, Osiris Property Explorer, and MolSoft toolkits [20,21,22,23].

## 2. Materials and Methods

### 2.1. Cell Culturing

Culturing of SH-EP1 cells [24] and stable transfection methods used to produce this cell line were described earlier [25]. Briefly, Dulbecco’s modified Eagle’s medium supplemented with 10% heat inactivated horse serum, 5% fetal bovine serum, 100 U/mL penicillin G, 100 µg/mL streptomycin, 0.25 µg/mL amphotericin B, 0.4 mg/mL hygromycin B, 0.25 mg/mL Zeocin, and 1 mM sodium pyruvate (all from Invitrogen, Carlsbad, CA, USA) were used to grow SH-EP1 cells on 35 mm dishes. Subsequently, cells were plated at a density of 2 × 10^5^ cells per well into 96-well plates and were held for 2–3 days in 5% CO_2_ saturated with H_2_O at 37 °C.

### 2.2. Intracellular [Ca^2+^] Measurements

These experiments were conducted as described earlier [9,14] at room temperature (24 ± 2 °C). Briefly, SH-EP1 cells were loaded with 10 µM fluo-4 AM (Molecular Probes, Life Technologies, Paisley, UK) in Krebs-HEPES solution (in mM: 144 NaCl, 5.9 KCl, 1.2 MgCl_2_, 2 CaCl_2_, 11 D-glucose, 10 HEPES, pH 7.4) for 45 min at 37 °C in the dark. Next, fluo-4 AM loaded cells were washed twice with Krebs-HEPES at room temperature. All test and incubation solutions contained atropine (1 µM). Fluorescence changes (excitation 485 nm, emission 520 nm) were measured using a fluorescent plate reader (Fluostar, BMG Labtech Inc., Cary, NC, USA). Changes in basal fluorescence levels were monitored before and after adding ACh containing solution through an automatic dispenser. Fluorescence changes were recorded for 30 s. The responses from each well were calibrated by measuring maximum and minimum fluorescence values to normalize fluo-4 signals. The Fmax value was obtained by the addition of 75 µL of 5% Triton X-100 and Fmin was attained by addition of 50 µL of 1 M MnCl_2_ at the end of the experiment. Data were presented as a percentage (%) of Fmax–Fmin or area under fluorescence curve (AUC). Apigenin, genistein, gossypetin, kaempferol, luteolin, phloretin, and quercetin were purchased from Sigma (Sigma, St. Louis, MO, USA). Flavonoids were dissolved in DMSO. At final concentrations of 0.01%, DMSO did not affect ACh-induced Ca^2+^ transient (*n* = 4).

### 2.3. Radioligand Binding Experiments

In 35 mm dishes, the SH-EP1 cells were grown to confluence, collected by scraping in 50 mM HEPES buffer solution containing 1 mM MgCl_2_, 2.5 mM CaCl_2_, 0.1% (*w*/*v*) bovine serum albumin, 0.025% (*w*/*v*) bacitracin, and 0.025% (*w*/*v*) sodium azide (pH 7.4), and centrifuged at 1200 r.p.m. for 15 min at 4 °C. Subsequently, the supernatant was removed and cells were frozen at −80 °C until the day of the experiment. For binding assays, using a Polytron tissue homogenizer at setting 4 for 20 s, the cells were resuspended in 50 mM Tris-HCl buffer containing 120 mM NaCl, 5 mM EDTA, 1.5 mM MgCl_2_, and 5 mM KCl (pH 7.4). In a total of 250 µL volume, 150 µL cell suspension, 50 µL radioligand [^125^I]-α-bungarotoxin (2200 Ci/mmol; Perkin-Elmer, Inc. Waltham, MA, USA) and 50 µL test compound, were added to 96-well microtitre plates. The α-bungarotoxin (3 µM) was used to determine non-specific binding. Subsequent to 45 min incubation at room temperature, the plates were filtered through Packard Unifilter-96, GF/C plates and washed twice with 500 µL ice-cold 10 mM Tris-HCl buffer containing 150 mM NaCl (pH 7.4). The radioactivity bound to filters was counted in 50 µL of scintillation solution (MicroScint 40, Perkin-Elmer, Inc. Waltham, MA, USA) in Packard TopCount scintillation counter. Assays were performed in triplicate.

### 2.4. Metric Parameters and Drug-Likeness Properties

Molecular weight (MW), water solubility (clogS), and Lipophilicity (clogP) of Lipinski’s rule for drug-likeness were calculated using the computational tool Osiris Property explorer. (Molinspiration software or free molecular property calculation services (last accessed 23 February 2021)) and Molinspiration property calculation toolkit [22,23]. The observed metric parameters for the tested polyphenol flavonoids are summarized in Table 1.

### 2.5. Statistical Analysis

The mean ± standard error means (S.E.M.) was used to present data. Statistical significance between measurements in different groups was determined using One-way ANOVA. When differences were found, pair-wise post-hoc comparisons using the Bonferroni correction were applied. The *p* values < 0.05 were considered significant. Radioligand saturation curves were obtained by fitting the data to the logistic equation, using non-linear hyperbolic curve fitting function of OriginPro 8.5 (OriginLab Corp., Northampton, MA, USA).

## 3. Results

**Figure 2:**
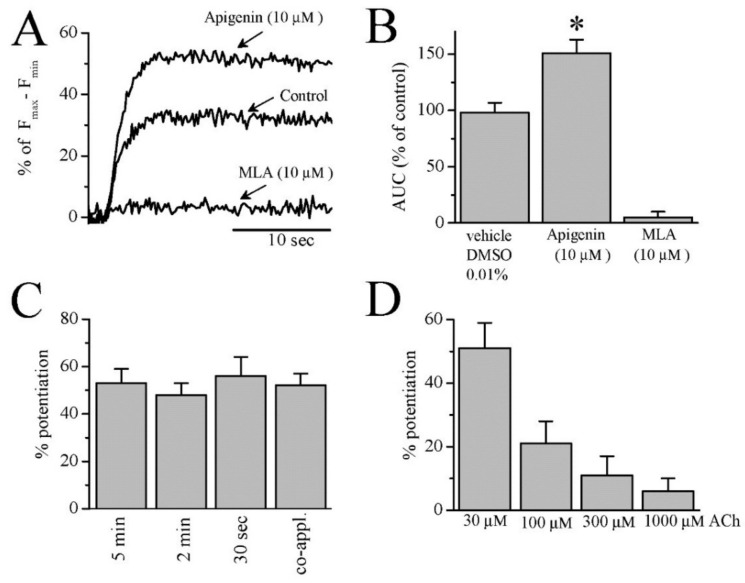
The effects of apigenin on Ca^2+^ transients elicited by the stimulation of human α7 nACh receptors expressed in SH-EP1 cells. (**A**) The effect of 10 µM apigenin and 10 µM methyllycaconitine (MLA) on Ca^2+^ transients induced by 30 μM ACh in 10 µM Fluo-4 AM loaded SH-EP1 cells. (**B**) Cumulative effects of apigenin and MLA on the area under curve (AUC) of Ca^2+^ transients induced by ACh. Bars indicate the mean ± S.E.M. * indicates *p* < 0.05 (ANOVA, *n*= 14–17). (**C**) The effect of preincubation time on the apigenin potentiation of Ca^2+^ transients induced by ACh (*n* = 9–12; ANOVA, *p* > 0.05). (**D**) The effect of increasing concentrations of ACh on the apigenin (10 µM) potentiation of ACh (30 μM)-induced Ca^2+^ transients. Bars indicate the mean % potentiation ± S.E.M. *n* = 11–15.

In preliminary experiments, no detectable changes in intracellular Ca^2+^ levels were observed after 30 s application of apigenin alone (up to 100 µM) in Fluo-4 loaded SH-EP1 cells (*n* = 11 from 3 separate experiments). On the other hand, rapid increases in intracellular Ca^2+^ concentrations were consistently observed following the application of 30 μM acetylcholine (ACh) (Figure 2A, control). These ACh-induced Ca^2+^ transients were completely inhibited after 5 min pre-incubation with methyllycaconitine (10 µM), a selective antagonist for α7-nACh receptor (Figure 2A).

Five min. pre-incubation of cells with 10 µM apigenin caused a significant potentiation (48% ± 5, *n* = 14, ANOVA, *p* = 0.001) of the ACh-induced Ca^2+^ transients (Figure 2A,B). Notably, a 5 min. application of 10 µM apigenin did not change the magnitude of the Ca^2+^ transient induced by the application of high-K^+^ (60 mM KCl, *n* = 11, ANOVA, *p* = 0.634), suggesting that the effects of apigenin are not due to the activity of voltage-dependent Ca^2+^ channels. Since most of the phytochemical effects were previously shown to be enhanced by the duration of the pre-application [8,9,10,11,12], we compared the effects of 5 min, 2 min, and 30 s apigenin pre-application, and also examined the effect of co-application of apigenin and ACh. The extent of apigenin potentiation of ACh-induced Ca^2+^ transient was found unchanged (Figure 2C) under various pre-application, as well as co-application time conditions. In earlier studies with curcumin, we observed that its potentiating effect was significantly diminished with increasing agonist concentrations [15]. Therefore, we tested the effect of increasing ACh concentration on apigenin potentiation of the Ca^2+^ transient. Interestingly, the effect of apigenin was significantly decreased at higher ACh concentrations (Figure 2D).

**Figure 3:**
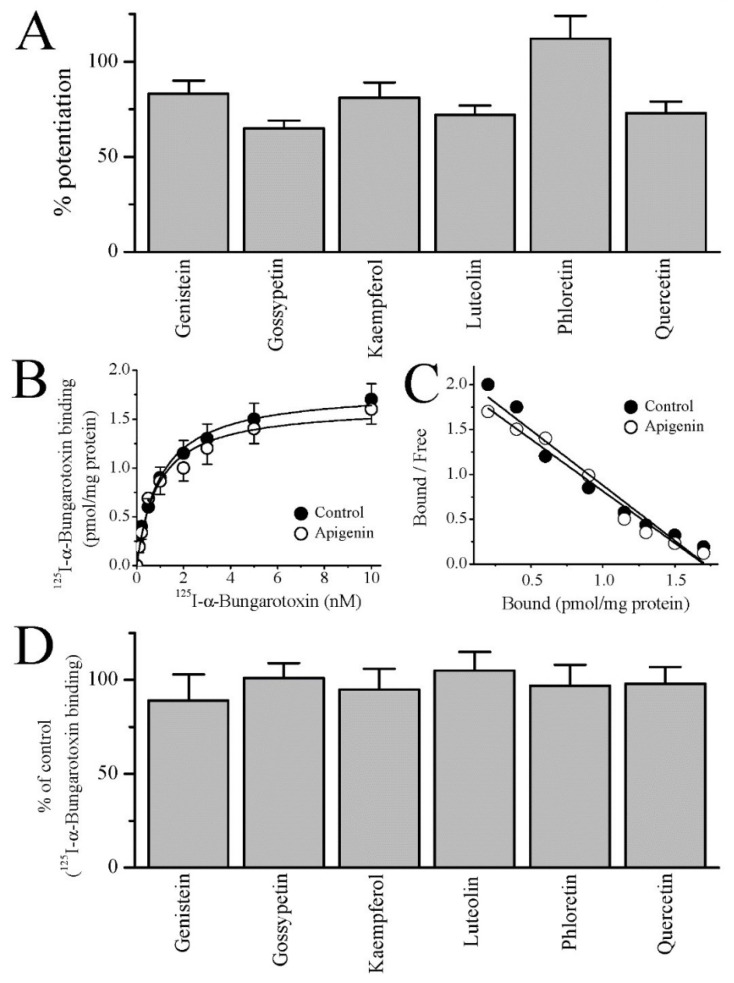
The effect of flavonoids on the Ca^2+^ transients induced by ACh and specific [^125^I] α-bungarotoxin binding in SH-EP1 cells. (**A**) The effect of 10 µM of genistein, gossypetin, kaempferol, luteolin, phloretin, and quercetin on ACh (30 μM)-induced intracellular Ca^2+^ transients. Bars indicate the mean ± S.E.M. *n* = 12–17. (**B**) The effect of apigenin on the binding saturation of [^125^I] α-bungarotoxin. Increasing concentrations of [^125^I] α-bungarotoxin are shown in X-axis as free ligand. SH-EP1 cells were incubated for 45 min. with the indicated concentrations of [^125^I] α-bungarotoxin in the absence (*filled circles*) and presence (*open circles*) of apigenin (10 μM). Unlabeled bungarotoxin (3 μM) was added to incubation buffer to determine non-specific binding (*n* = 4–6) (**C**) Scatchard analysis, apigenin effects on saturation binding of [^125^I] α-bungarotoxin. Units are fmol/mg protein and fmol/mg protein/nM for x and y axis, respectively. (**D**) Effects of flavonoids on the specific binding of 2 nM [^125^I] α-bungarotoxin in the same cell line. Bars indicate the mean ± S.E.M. *n* = 9–12.

Next, we investigated the effects of the other polyhydroxy flavonoids genistein, gossypetin, kaempferol, luteolin, phloretin, and quercetin on ACh-induced Ca^2+^ transients in the same cell line. No detectable changes in intracellular Ca^2+^ levels were observed after 30 s applications of these flavonoids alone (up to 30 µM) in Fluo-4 loaded SH-EP1 cells (*n* = 7–12 from 4 separate experiments). At 10 μM, all flavonoids tested caused a significant potentiation of ACh (30 μM)-induced Ca^2+^ transients (Figure 3A), with a potency profile of: phloretin (112% ± 12) > genistein (83% ± 7) ≥ kaempferol (81% ± 8) > quercetin (73% ± 6) ≥ luteolin (72% ± 5) > gossypetin (65% ± 4).

In subsequent studies, we investigated the effect of apigenin on specific binding of [^125^I] α-bungarotoxin, a competitive antagonist of ACh at the α7-nACh receptor [1]. Saturation curves for [^125^I] α-bungarotoxin binding in the absence (controls) and presence of apigenin are shown in Figure 3B. In SH-EP1 cells preincubated (45 min) with 10 μM apigenin, there was no significant change in [^125^I] α-bungarotoxin binding. The apparent affinity (K_D_) of the receptor for [^125^I] α-bungarotoxin was 1.18 ± 0.29 and 1.03 ± 0.32 pM for controls and apigenin, respectively (*n* = 14 in 3 experiments; ANOVA, *p* = 0.097). In line with this finding, Scatchard analysis of saturation binding data indicated that Bmax values in the absence and presence of apigenin (10 µM) were not changed significantly (Figure 3C). The Bmax values were 1.73 ± 0.09 pmol/mg in controls and 1.71 ± 0.11 pmol/mg in the presence of apigenin (*n* = 11 measurement from 3 experiments, ANOVA, *p* = 0.086). Finally, we tested the effects of 10 μM genistein, gossypetin, kaempferol, luteolin, phloretin, and quercetin on [^125^I] α-bungarotoxin binding. Similarly to apigenin, these polyhydroxy flavonoids did not change [^125^I] α-bungarotoxin binding in SH-EP1 cells (Figure 3D).

## 4. Discussion

In the present study, we provide evidence that flavonoids, such as apigenin, genistein, gossypetin, kaempferol, luteolin, phloretin, and quercetin, allosterically potentiate human α7-nACh receptors expressed in SH-EP1 cells. In addition, we present some important physicochemical parameters suggested to be useful in selecting oral drug candidates to facilitate drug discovery and development processes [22,26]. In this context, water-solubility (clogS), lipophilicity (clogP), molecular weight (MW), drug-likeness score, and topological polar surface area (TPSA), parameters closely related to Lipinski’s rule, were calculated for the current panel of tested compounds (Figure 1) by applying the Osiris Property explorer and the Molinspiration property calculation toolkit (Table 1) [26,27]. The clogS value indicating the drug solubility affects its absorption and distribution properties. Accordingly, the solubility of the tested compounds was found in an acceptable range (<−4 clogS). In addition to solubility, drug-likeness scores and the lipophilicity-related physicochemical parameters, such as clogP, have been shown to modify drug potency, pharmacokinetics, and toxicity, and are recognized as useful tools in the lead optimization process [21,23,26]. Consequently, ligands with a clogP < 5 were suggested to present more promising drug-likeness profile [28,29]. Among the current panel of tested compounds, the clogP values were calculated as <5 suggesting the suitability of the compounds for oral administration (Table 1). The TPSA values have also been used in development of a successful drug candidate. In general, compounds with TPSA values > 60 Å^2^ are considered poorly membrane-permeable molecules with relatively decreased CNS bioavailability [22,26]. Among the tested polyphenol flavonoids, the calculated TPSA values were in the range of 77–131 Å^2^, suggesting physicochemical parameters expected from drug-like compounds, especially regarding TPSA (Table 1). Another numerical value useful in drug development process is the drug-likeness model score which signifies a combined result of physicochemical, pharmacokinetic, and pharmacodynamic properties of the compound [22]. Thus, ligands having zero or negative values are considered less suitable as a drug-like candidate. In this study, all flavonoids, except for phloretin, have drug-likeness scores in the range of 0.67–1.91; with apigenin, luteolin, and quercetin showing maximum-likeness scores of 1.12, 1.91, 1.64, respectively (Table 1; Figure 1).

Preincubation with apigenin did not alter the extent of its effect, and co-application with ACh was sufficient for potentiation of ACh-induced Ca^2+^ transients, suggesting that membrane partitioning and/or the phosphorylation of the α7-nACh receptor are not required for the observed effect. Importantly, positive modulatory effect of apigenin was significantly diminished by increasing concentrations of ACh. It is possible that desensitized α7-nACh receptors at high ACh concentrations have lower affinity to apigenin. In line with this hypothesis, after complete desensitization of α7-nACh receptors with (100 μM ACh for 1 min), apigenin failed to potentiate Ca^2+^ transients (data not shown, *n* = 3).

Moreover, other polyhydroxy flavonoids tested also potentiated ACh-induced Ca^2+^ transients with potency order of genistein > gossypetin > kaempferol > luteolin, phloretin, and quercetin. Radioligand binding experiments indicate that apigenin and other flavonoids does not alter [^125^I] α-bungarotoxin binding suggesting that these compounds act as allosteric modulators of the α7-nACh receptor. These results confirm earlier findings with genistein and quercetin [17,18,19], and identify apigenin, and other flavonoids, as likely PAM of the human α7-nACh receptor. The PAMs are promising therapeutic agents since they maintain the temporal and spatial characteristics of the endogenous activation of the receptor and are usually more selective than agonists [1].

Combining these results, apigenin and structurally related other polyhydroxy flavonoids revealed promising drug-likeness values, and underlined a role for polyphenol flavonoids in the regulation of α7-nACh receptor signaling and their potential clinical use in conditions ranging from the treatment of pain and inflammation to alleviating neurodegenerative disorders.

## Figures and Tables

**Table 1:** Drug-likeness calculations and Lipinski parameters for tested polyphenol flavonoids. ^a^ molecular weight, ^b^ topological polar surface area, ^c^ water solubility (clogS), ^d^ lipophilicity (clogP), ^e^ Molinspiration software or free molecular property calculation services (Molinspiration software or free molecular property calculation services (last accessed 23 February 2021)).

Compound	MW ^a^	TPSA ^b^	cLogS ^c^	cLogP ^d^	Drug-Likeness Model Score ^e^
Apigenin	270.24	90.89	−2.86	2.34	1.21
Genistein	270.24	86.99	−2.73	1.63	1.16
Luteolin	286.24	111.12	−2.56	1.99	1.91
Kaempferol	286.24	107.21	−2.79	1.84	0.91
Quercetin	302.24	131.35	−2.49	1.49	1.64
Gossypetin	318.24	127.42	−2.19	1.14	0.67
Phloretin	258.27	77.75	−2.52	2.04	−0.56

## Data Availability

All data are included in the manuscript.
